# A relook into plant wilting: observational evidence based on unsaturated soil–plant-photosynthesis interaction

**DOI:** 10.1038/s41598-020-78893-z

**Published:** 2020-12-16

**Authors:** Ankit Garg, Sanandam Bordoloi, Suriya Prakash Ganesan, Sreedeep Sekharan, Lingaraj Sahoo

**Affiliations:** 1 Guangdong Engineering Center for Structure Safety and Health Monitoring, Shantou University, Guangdong, China; 2 Department of Civil and Environmental Engineering, The Hong Kong University of Science and Technology, Hong Kong, SAR China; 3 Department of Civil and Environmental Engineering, Shantou University, Guangdong, China; 4 Department of Civil Engineering, Indian Institute of Technology, Guwahati, Assam India; 5 Department of Biosciences and Bioengineering, Indian Institute of Technology, Guwahati, India

**Keywords:** Plant sciences, Hydrology, Engineering

## Abstract

Permanent wilting point (PWP) is generally used to ascertain plant resistance against abiotic drought stress and designated as the soil water content (θ) corresponding to soil suction (ψ) at 1500 kPa obtained from the soil water retention curve. Determination of PWP based on only pre-assumed ψ may not represent true wilting condition for soils with contrasting water retention abilities. In addition to ψ, there is a need to explore significance of additional plant parameters (i.e., stomatal conductance and photosynthetic status) in determining PWP. This study introduces a new framework for determining PWP by integrating plant leaf response and ψ during drought. *Axonopus compressus* were grown in two distinct textured soils (clayey loam and silty sand), after which drought was initiated till wilting. Thereafter, ψ and θ within the root zone were measured along with corresponding leaf stomatal conductance and photosynthetic status. It was found that coarse textured silty sand causes wilting at much lower ψ (≈ 300 kPa) than clayey loam (≈ 1600 kPa). Plant response to drought was dependent on the relative porosity and mineralogy of the soil, which governs the ease at which roots can grow, assimilate soil O_2_, and uptake water. For clay loam, the held water within the soil matrix does not facilitate easy root water uptake by relatively coarse root morphology. Contrastingly, fine root hair formation in silty sand facilitated higher plant water uptake and doubled the plant survival time.

## Introduction

In the wake of climate change and population growth (9.2 billion by 2050 as per U.N. Population Division 2008), water supply to sustain the agriculture sector has been predicted to face a severe shortage, especially in arid regions^1,2^. A rise in global temperature (by 0.5 °C in the past 100 years^3^), coupled with a gradual decrease in atmospheric precipitation has resulted in higher vulnerability of plant to drought stresses^4,5^. Soil water availability has also been predicted to partake in a decreasing trend as per model studies based on the International Panel on Climate Change (IPCC) estimates^6^. Soil water retention curve (SWRC) is commonly used to indicate the soil’s ability to hold water and is the relationship between the soil water content (θ) and the soil water potential commonly referred as soil suction (ψ)^7^. SWRC is commonly used to represent the moisture status of the root zone in vegetated soil^8^. In addition with SWRC, one of the other key parameters that govern irrigation schemes and vegetation survival is permanent wilting point (PWP). PWP is defined as the percentage amount of water per unit weight or bulk volume of the soil that cannot be absorbed by plant roots^9^. According to the classic definition given by Briggs and Shantz^10^, PWP was originally defined as “the moisture content of the soil at which the leaves of the plant undergo a permanent reduction in their moisture content as a result of a deficiency in the soil-moisture supply”. Based on the classical definitions, it is understood that the PWP is directly dependent on the plant’s ability to withdraw water from the soil matrix, and the parameter should ideally not have a unique value.

In the past decades, soil science practitioners, agriculturists, and ecologists have determined PWP from the SWRC of vegetated soil^11,12^ as the soil water content corresponding to a soil matric potential of 4.2 pF (i.e., 1500 kPa). This reference value probably was suggested based on the measurement limit of the pressure plate apparatus up to pF 4.2, which was the most popular instrument for measuring soil suction post 1950^13^. Another plausible reason could be the popularity of using crop-growth models, which necessitated a maximum limiting suction value corresponding to PWP. The knowledge of PWP is essential in designing irrigation schemes for drought-prone areas^14^, ecological rehabilitation^15^, and urban green infrastructure^16^. Improper determination of PWP without considering the plant physiology might result in over-irrigation, leading to excess water loss by evaporation and deep drainage. On the contrary, under irrigation may lead to plant wilting, especially in coarse grained soil. Therefore, the approach of determining PWP at 1500 kPa as an invariant index representing the lower limit of plant water availability may be misleading^17^. Kirkham has criticized that PWP at 1500 kPa overestimates the water availability for plants in case of fine-textured (clay) soils where sufficient bound water may be present, which cannot be extracted by plant roots. On the contrary, for coarse-textured (sand) soils, the soil reaches residual water content (2–3%) even at (200–500) kPa^18^. Considering the extreme events due to climate change such as prolonged droughts, limited irrigation water availability, and species prone to drought stress, it is important to re-look into the quantification of PWP.

**Figure 1:**
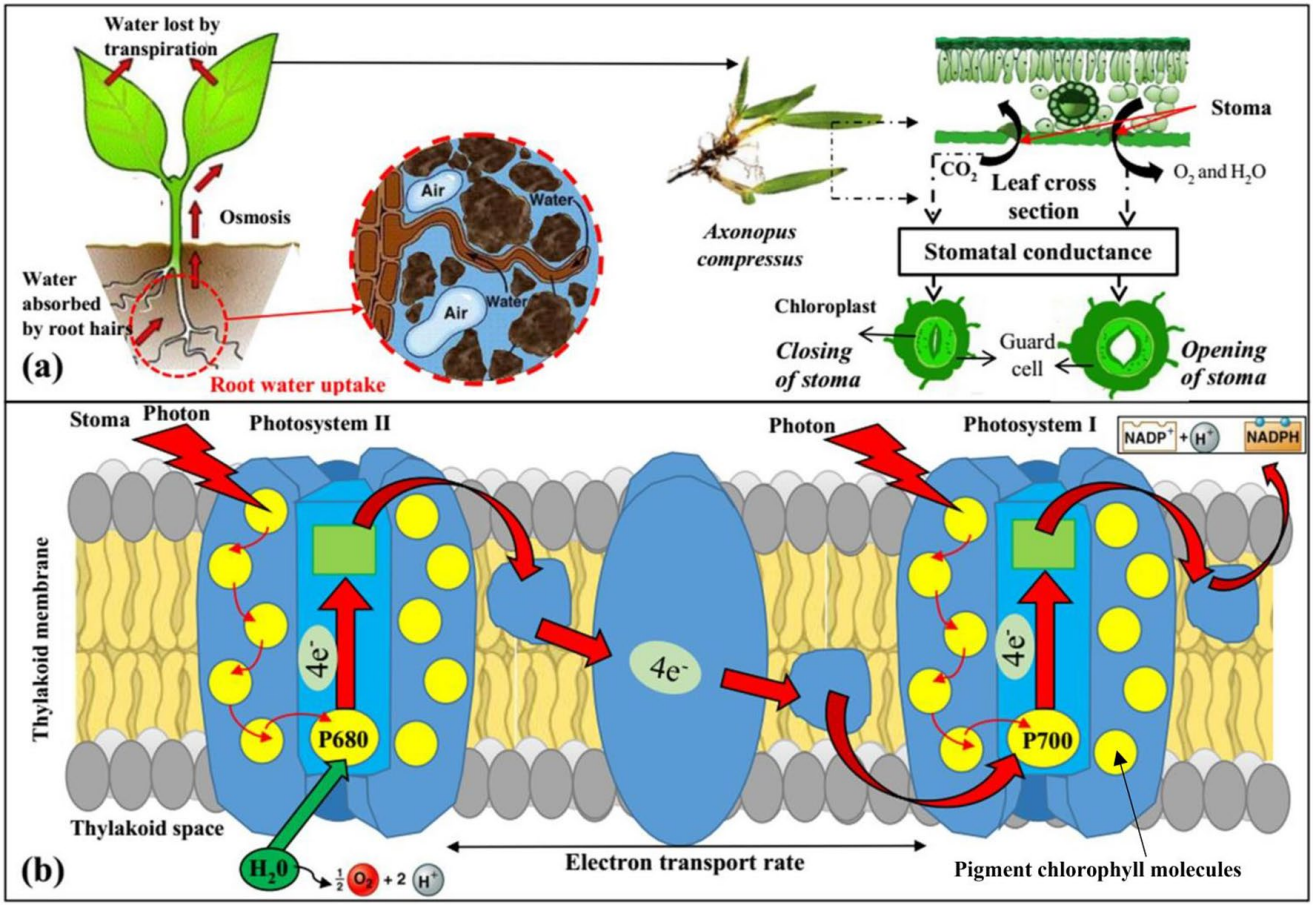
Schematic diagram depicting (**a**) stomatal functioning, initiated by transport of root water uptake and nutrient to plant leaf. The steps in sequence represents transpiration mechanism, indicated by loss of water via opening and closure of stomata across the leaf section; (**b**) light dependent photosynthesis process. Photosystems (groups of photosynthetic pigments (including chlorophyll pigment molecules) embedded within the thylakoid membrane) absorbs light energy to energize the delocalized electrons. The energized electrons are transferred to carrier molecules within the thylakoid membrane (electron transfer chain) for further ATP production (phosphorylation) and reduction of NADP+ .

Inherent plant leaf parameters (refer Fig. 1) such as stomatal conductance (SC)^19^ and photosynthesis parameters^20^ have been found to be sensitive to leaf water potential, root zone moisture, and soil suction. Tezara et al.^21^ explored drought sensitivity for *Helianthus annuus* and reported that water stress affects adenosine triphosphate synthase and results in decreased CO_2_ assimilation. On the other hand, stomatal closure was also observed as a coping mechanism for plants to inhibit drought stress associated with the release of root chemicals^22^, loss of leaf turgor, and low humidity^23^. In all these established studies, the role of soil suction within the root zone, indicating the status of root water uptake, was usually ignored. Stomatal pores in the leaf surface progressively close during drought stress, decreasing the SC, and thereby slowing the transpiration rate^24^. Photosynthetic parameters have also been extensively reported to change its functioning with the change in season or in environmental conditions^25^. Not many studies investigated the additional role of shoot/root biomass adaptivity on wilting by linking the plant leaf parameters with soil suction. Such a holistic approach can provide observational evidence to identify wilting susceptibility and formulate reliable and efficient irrigation schedule under water stressed condition.

**Figure 2:**
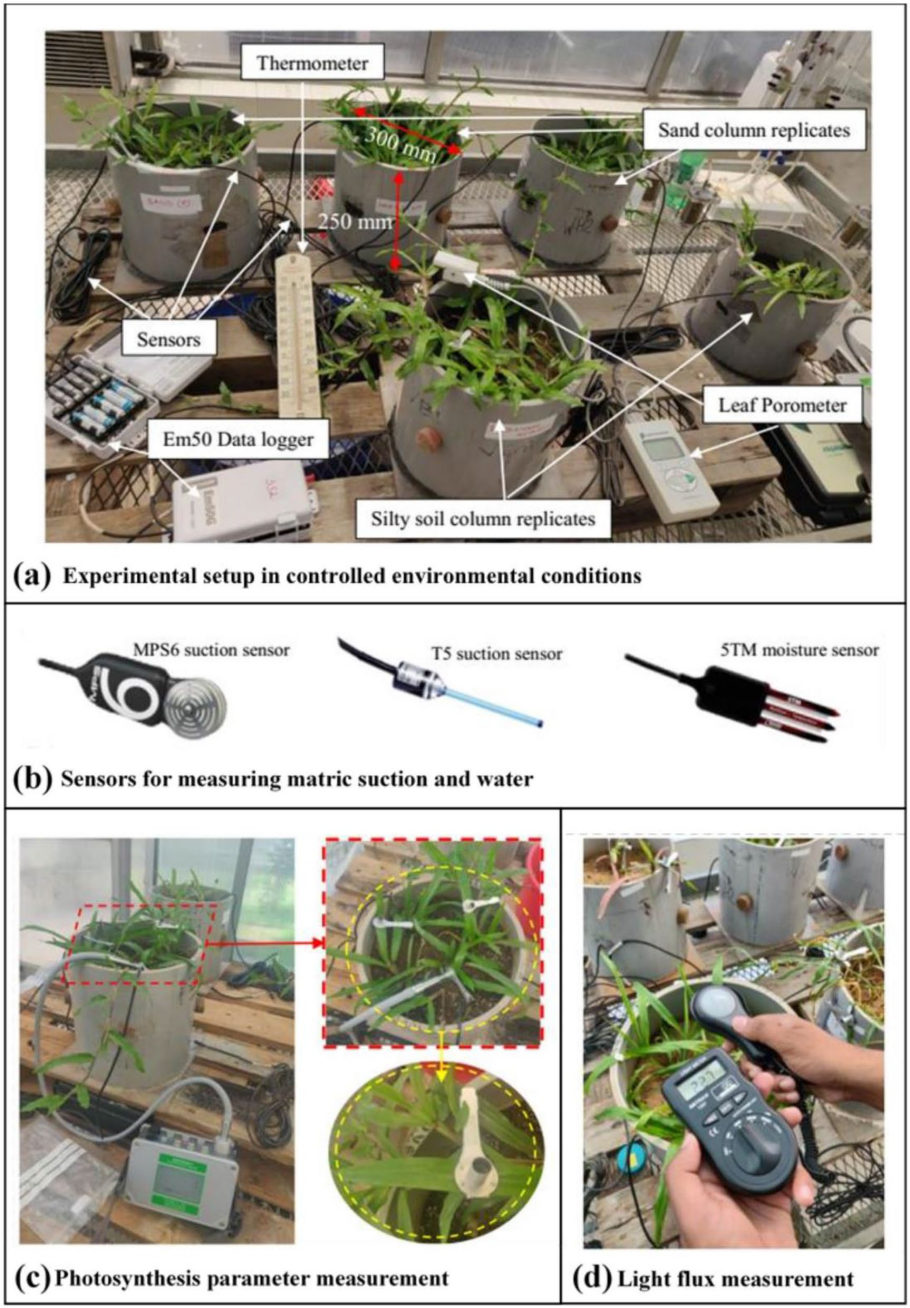
Pictorial presentation of (**a**) Vegetated columns with instrumentation to monitor soil hydrological parameters, stomatal conductance and in-house temperature; (**b**) sensors used in the study to measure matric water potential (ψ) and volumetric water content (θ) of the soil medium; (**c**) photosynthesis measurement setup using DLC-8 dark leaf clip and MINI-PAM-II; (**d**) ensuring the optimal light intensity for plant growth using lux meter.

This study attempts to explore the wilting susceptibility of a native plant species, common to South-East Asia. The main objective of this study is to develop a new approach or framework for determining the permanent wilting point of plant species by integrating plant leaf and soil characteristics during drought. Two sets (with three replicates) of column studies were performed wherein *Axonopus compressus* (a native grass species from South Asia, Fig. 2a) were grown in two contrasting soils (silty sand and clayey loam). The plants were grown in uniformly compacted soil for two months, after which drought was initiated till wilting. The soil matric suction (ψ) and volumetric water content (θ) within the root zone were measured (Fig. 2b) along with the corresponding leaf photosynthetic parameters (SC, photosynthetic efficiency, maximum CO_2_ fixation rate, maximum photosynthetic yield, and effective photosynthetic yield).

## Materials

### Selection of soil and plant

Two soils with contrasting water retention abilities were selected in this study from the state of Assam, India. Silty sand represents coarse soil with 56% sand fraction followed by silt and clay at 41% and 3%, respectively. The other soil was a clayey loam with 47% sand, 28% silt, and 21% clay fraction. The specific gravity of the silty sand and clayey loam is 2.65 and 2.69, respectively. The maximum relative compaction density of each soil was found at 1.7 g/cc and 1.68 g/cc, respectively. The other basic index properties of soil are listed in Table 1. It is important to emphasize that the soils were collected in areas that had been subjected to severe droughts in the past. The plant species selected for the study is *Axonopus compressus*, which is a common pasture grass for cattle rearing and used for bioengineering of slopes in South-East Asia. *Axonopus compressus* was selected based on its easy availability, drought tolerance, and use in ecological restoration measures^26^. This species is native to the Aravalli Range for both types of soils used in the current study.

**Table 1:** Basic soil properties measured in the current study.

Material properties	Clay loam	Silty sand
**Particle size distribution**		
Gravel (> 4.75 mm) (%)	0	0
Coarse sand (2.00–4.75 mm) (%)	0	0.04
Medium sand (0.425–2.00 mm) (%)	7	0.28
Fine sand (0.075–0.425 mm) (%)	18	98.50
Silt (0.002–0.075 mm) (%)	50	1.18
Clay (< 0.002 mm) (%)	25	0
**Atterberg limits (ASTM D4318-00)**		
Liquid limit (%)	36.5	–
Plastic limit (%)	25.6	–
**Compaction parameters (ASTM D698-07)**		
OMC (%)	16.4	–
MDD (g/cc)	1.71	1.72
Specific gravity (ASTM D854-14)	2.65	2.69
pH	6.85	7.6
**Agricultural properties**		
CEC	8	7.3
Organic content	2.48	1.31
Total N (ppm)	3.6	0.31
Total P (ppm)	1.8	1.4
Total K (ppm)	3	1.9
USCS classification*	ML	SP

*Unified soil classification system.

### Instrumentation

Three different sensors were used in this study to measure soil suction and soil water content. The TEROS-21 sensor measures soil matric suction by determining the dielectric permittivity and water content of a porous ceramic disk, which is in equilibration with the surrounding soil. The circuit board in TEROS-21 comprises an oscillator that generates an electromagnetic field during suction measurement. The electromagnetic field charges the ceramic disk, and the stored charge measures the dielectric permittivity and water content of the ceramic disks. From the ceramic water content, matric suction was determined based on the moisture characteristic curve of the porous ceramic. It should be noted that the generated electromagnetic field is mainly confined to the ceramic disk of the sensor so that the measurements taken are independent of soil type. The sensor has an operating range of 9 kPa to 100,000 kPa with a measurement speed of 150 ms^27^.

The tensiometer (T5 small tip tensiometers) also measures soil suction and consists of a tensiometer body, shaft, and a ceramic cup. The tensiometer body is threaded to a transparent acrylic shaft, which is filled with distilled water free from any air bubbles. The other end of the shaft is connected to a tensiometer ceramic cup. When the tensiometer is inserted in soil for measurement, the water present in the shaft tries to equilibrate with the moisture present in the soil via a porous ceramic interface with known air-entry value. The changes in water tension of the shaft are captured electronically and converted to pressure^28^. The volumetric water content sensor, 5TM measures soil water content. The measurement methodology of this instrument is based on the dielectric permittivity of the soil. The value for dielectric permittivity of water is 80, while for dry soil and air, it is around 4 and 1, respectively. This broad range of permittivity helps in measuring the soil water content from saturated to dry state conditions. 5TM measures the dielectric permittivity (in terms of raw data) value, which is thereafter converted to soil volumetric water content^29^.

## Methods

### Experimental procedure

All vegetated columns were grown in a greenhouse, wherein the temperature and humidity were controlled at 30 ± 3 °C and 50 ± 4%, respectively (Fig. 2a)^30^. To provide radiant energy for plant growth, fluorescent lights with light intensity 50 ± 10 µmol m^−1^ s^−1^ were provided at 2.5 m height from the column surface. The light intensity provided is in accordance with the daily light integral (DLI) requirements and are adequate for plant growth^31,32^. The soil was then compacted in a cylindrical polyvinyl chloride (PVC) column of dimension (300 mm diameter, 250 mm height) and vegetated with non-crop species (*A. compressus*). Three rhizomes having equal biomass were transplanted in each column of the test (6 columns in total, considering three replicates for both soil type). The rhizome transplanted columns are instrumented with soil suction measuring sensors and volumetric water content sensors. All the sensors were embedded at a depth of 40 mm below the ground surface, ensuring measurements were within the root zone (major root proportions seen after excavation)^33,34^. Vegetation grew in the soil medium for the next 60 days. Prior to imposing drought on the soils, the soil was regularly watered (ponded) such that water percolation through the bottom perforated plate was observed. From the moisture sensor reading, it was seen that water ponded was enough to keep the root zone near field capacity, i.e., close to saturated water content (θs). After growth corresponding to 60 days, drought stresses were applied by restricting any further irrigation on the vegetation until they wilted. The unsaturated soil parameters (i.e., ψ, θ) were measured and recorded in the EM-50 data logger. To eliminate any loss of data, sensor readings were stored in the data logger with a logging interval of 10 min.

The plant leaf parameters such as SC and photosynthetic responses were measured simultaneously using the leaf porometer (SC-1, Meter Inc. USA) and MINI-PAM-II^35^, respectively. The desiccant used in the leaf porometer was regularly changed and calibrated using deionized water for reliable measurements of plant leaf SC. For photosynthetic measurements, the analysis was made using red light illumination with the wavelength ranging between 600 and 700 nm, as the chlorophyll absorbs red light effectively. For dark adapted examinations, the leaves were covered using DLC-8 dark leaf clip for easier positioning of fiber optics (Fig. 2c). The measured parameters were analyzed and plotted against corresponding soil suction values. Leaf water contents at different stages of drought stress were also provided in Table 2. During this monitoring period, the light intensity of the greenhouse was constantly cross-checked using a light flux meter (Fig. 2d). Statistical analysis in the form of mean and percentage error was performed, and normalized error percentages are summarized in Tables 2 and 3. The total plant biomass at the end of the experiment was calculated by cutting the dried shoots and excavated roots.

**Table 2:** Summary of measured parameters and associated statistical analysis at different stages of drought.

Different stages of drought	Parameters	Clay loam columns					Silty sand columns				
		1	2	3	Mean	% error	1	2	3	Mean	% error
**Boundary effect zone** *Suction values* Clay loam—11.9 kPa Silty sand—5.1 kPa	Soil water content	32.9	34.5	32.7	33.4	2.26	44.6	46.8	47.4	46.3	2.37
	Stomatal conductance	147.1	134.9	162	148.0	6.30	198	167	177	180.7	6.39
	Photosynthetic efficiency	0.0342	0.0326	0.0373	0.0347	5.00	0.0598	0.0521	0.0548	0.055	5.08
	Maximum CO_2_ fixation rate	5.26	5.03	5.76	5.35	5.09	14.13	12.78	14.49	13.80	4.92
	Maximum yield	0.734	0.744	0.720	0.733	1.15	0.772	0.789	0.764	0.775	1.20
	Effective yield	0.210	0.193	0.219	0.207	4.61	0.475	0.426	0.433	0.445	4.54
	Leaf water content	0.79	0.81	0.83	0.81	1.64	0.80	0.83	0.84	0.823	1.88
**Transition zone** *Suction values* Clay loam—284.3 kPa Silty sand—8.6 kPa	Soil water content	30.4	31.7	32.4	31.5	2.33	18	18.9	17.8	18.2	2.44
	Stomatal conductance	86.8	76.1	89.1	84	6.26	128	136.1	113.7	125.9	6.49
	Photosynthetic efficiency	0.038	0.034	0.037	0.036	5.01	0.0582	0.0497	0.0547	0.054	4.92
	Maximum CO_2_ fixation rate	5.73	4.93	5.33	5.33	4.99	12.18	10.93	12.32	11.81	4.96
	Maximum yield	0.733	0.747	0.722	0.734	1.08	0.741	0.757	0.764	0.754	1.15
	Effective yield	0.287	0.254	0.263	0.268	4.72	0.469	0.412	0.433	0.438	4.71
	Leaf water content	0.72	0.73	0.75	0.733	1.52	0.75	0.72	0.74	0.737	1.51
**Residual zone** *Suction values* Clay loam—1365 kPa Silty sand—108.2 kPa	Soil water content	20.8	21.6	22.3	21.57	2.37	1.15	1.08	1.13	1.12	2.38
	Stomatal conductance	36.5	38.1	32.2	35.60	6.36	37	36.6	31.7	35.1	6.45
	Photosynthetic efficiency	0.0102	0.0112	0.0098	0.0104	5.12	0.0251	0.0246	0.0221	0.023	5.10
	Maximum CO_2_ fixation rate	0.72	0.80	0.71	0.74	4.85	3.76	3.46	3.98	3.73	4.88
	Maximum yield	0.184	0.186	0.180	0.183	1.21	0.616	0.632	0.612	0.62	1.29
	Effective yield	0.036	0.037	0.041	0.038	4.91	0.164	0.152	0.144	0.153	4.64
	Leaf water content	0.62	0.59	0.61	0.61	1.83	0.60	0.62	0.63	0.62	1.62

**Table 3:** Standard error percentage considered for plotting the graphs.

Parameters	Normalized % error
Soil water content (%)	2.5
Stomatal conductance (mmol m^−2^ s^−1^)	6.5
Photosynthetic efficiency	5.0
Maximum CO_2_ fixation rate (μmol CO2 m^−2^ s^−1^)	5.0
Maximum yield	1.5
Effective yield	5.0
Leaf water content	2.0

### Measurement of stomatal condutance and photosynthetic parameters

The changes in vapor transport through the process of stomatal closure and opening is measured by a parameter termed SC. SC is the measure of water vapor exiting or rate of CO_2_ passage through the stomata of the leaf. It is a function of stomatal density, size, and aperture and is an indirect measure of transpiration and gas passage. The photosynthetic status is measured using the principle of “Chlorophyll fluorescence” which acts as an indicator for photosynthetic energy conversion^36^. This phenomenon was first observed by Kautsky et al.^37^ and explains the basic photosystem reaction on photon illumination. The Kautsky effect described that when a dark-adapted leaf was subjected to photon illumination, the chlorophyll fluorescence variation was due to photochemical quenching (photons used for electron transport in photosynthesis process) or non-photochemical quenching (photons converted for heat dissipation). Zhu et al.^38^ additionally elucidated that this variation in chlorophyll fluorescence was only due to Photosystem II (PS-II) reaction center as the chlorophyll fluorescence is constant in PS-I. In the context of chlorophyll fluorescence, rapid light curve and induction curve are majorly used to study the photosynthetic quantum efficiency of the plant leaf.

**Figure 3:**
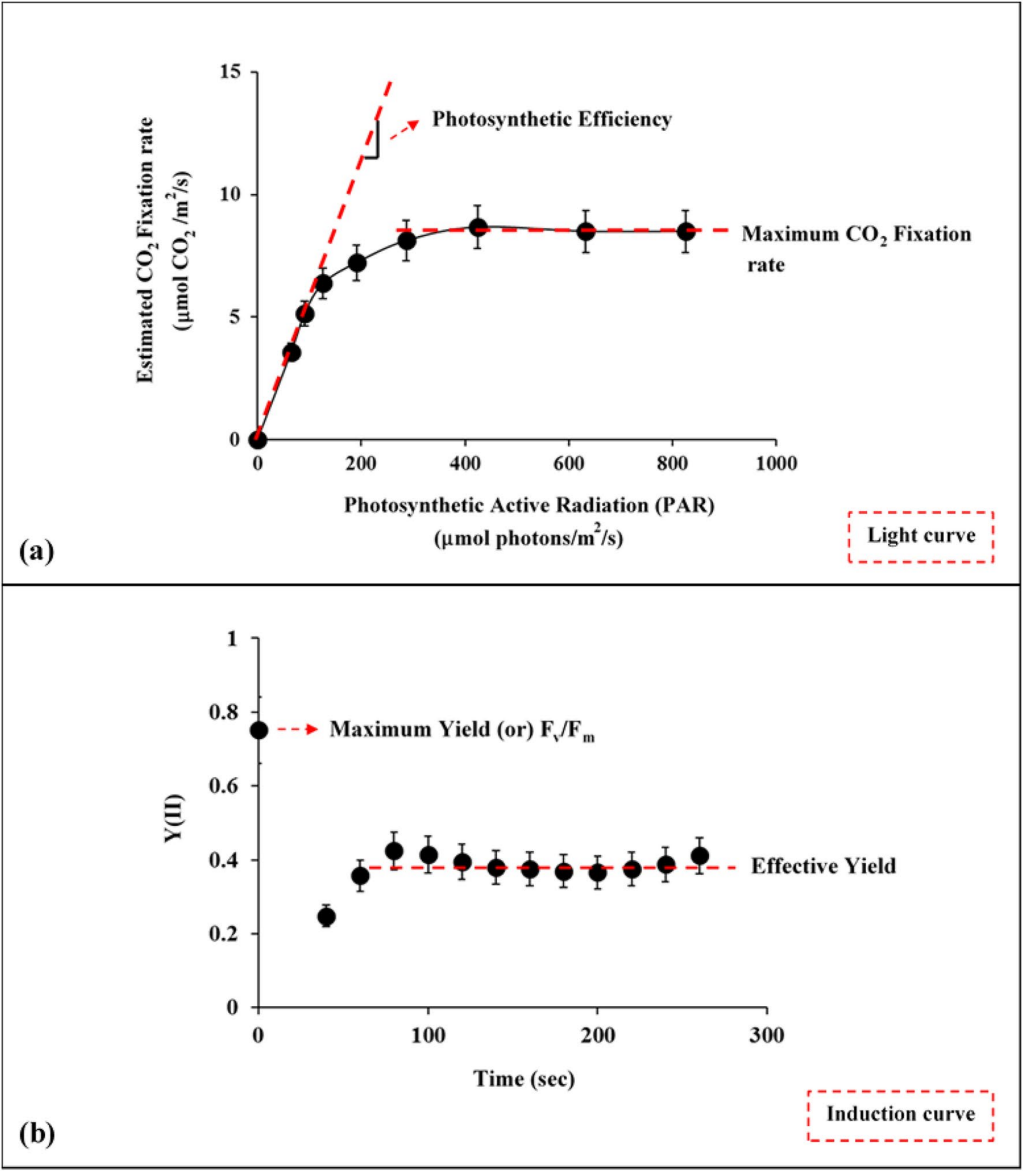
Typical photosynthetic parameters derived from (**a**) light curve and (**b**) induction curve. The curves were obtained directly from MINI-PAM-II window settings and are individually analyzed to derive the parameters.

Rapid light curves measure the electron transfer rate (ETR) with an increase in light intensity. The light intensity is expressed as photosynthetic active radiation (PAR), which is a measure of the light of wavelengths between 400 and 700 nm. To compute the light curve, leaves are initially dark acclimated for 15 min, thereby assuring that the photosystem reaction centers PS(II) are opened^39^. After dark acclimation of leaves, light intensities ranging between 0 to 900 PAR values were illuminated at 9 intervals, and the corresponding ETR values were measured. The ETR value for a particular light intensity is obtained as a single output from the following equation^40^

1 $$\text{ETR}=\text{PAR}*\text{ETR factor}* 0.5 *\text{Y}(\text{II})$$

where, PAR is the quantum flux density of photosynthetic active radiation acting on the leaf; ETR factor is the sample absorbance value (default value is 0.82 whereas the value will be lesser for dried leaves). The discussions regarding the measurements of Y(II) are elaborated in the later section. From the attained ETR values, the rate of CO_2_ fixation rate could be estimated, considering that 4 mol of electrons are required for fixing 1 mol of CO_2_^41–43^. This estimation is carried out mainly because the relation between PAR and net CO_2_ assimilation determines the extent of the photorespiration^44^. Figure 3a depicts the typical light curve of the estimated CO_2_ fixation rate with an increase in light intensity. In the figure, there is a clear trend that the CO_2_ fixation rate approaches a constant value after certain light intensity due to a decrease in the values of Y(II) and ETR at higher PAR values. Moreover, beneath certain light intensity, the reaction centers are assumed to be closed as it cannot accept any further photons^38^. Figure 3a also represents the parameters analyzed from the light curve, i.e., photosynthetic efficiency and maximum CO_2_ fixation rate. The first parameter, ‘photosynthetic efficiency’ explains the efficacy of the photosynthesis, i.e., the effective conversion of light into energy molecules, which is obtained by assessing the slope of the linear phase of the response curve^43^. The other parameter ‘maximum CO_2_ fixation rate’ is estimated by the average of CO_2_ fixation rate per unit cell at higher PAR values^45^.

Figure 3b depicts the induction curve representation of the yield value (Y(II)) or the light absorption capacity of the photosystem-II at different time intervals. In induction curve measurements, minimum fluorescence (F_0_) and maximum fluorescence (F_m_) are simultaneously measured in both dark and light adapted state. These measurements are essential to understand the status of chloroplast metabolism, which equips the pathways for carbon assimilation depending on the dark and light adapted conditions^46^. Two significant parameters F_v_/F_m_ and Y(II) are determined from the induction curve to represent dark and light adapted metabolic status, respectively.

As seen from Fig. 3b, both F_v_/F_m_ and Y(II) are measured consecutively with delayed time duration. The ratio F_v_/F_m_ is determined only in a dark-adapted leaf sample, such that the reaction centers are opened, and there will be minimal non-photochemical dissipation. After dark acclimation, measuring light intensity was illuminated to obtain minimum fluorescence (F_0_). This is followed by sending a sudden saturation pulse, which closes the reaction centers for measuring maximum fluorescence (F_m_). Through the measured fluorescence values, the F_v_/F_m_ ratio is calculated by:

2 $$\frac{{F}_{V}}{{F}_{m}}=\frac{{F}_{m}-{F}_{0}}{{F}_{m}}$$

The ratio measured in the dark-adapted leaf provides the maximum light absorption capacity or maximum yield (F_v_/F_m_) of PS-II reaction center^47^. After measuring the ratio F_v_/F_m_, far red light with a frequency above 700 nm is illuminated to open the closed reaction centers. Ideally, it takes only 5 s of illumination. Later, the leaf is exposed to steady-state photosynthetic lighting conditions for measuring Y(II)^48,49^. The maximum fluorescence (F_m′_) is similarly measured by sending a saturation pulse, which also closes the reaction centers. A fluorescence level (F) is measured shortly before the application of a saturation pulse. This F represents the momentary fluorescence in a light adapted state. The effective quantum efficiency, Y(II) is measured using:

3 $$Y\left(II\right)=\frac{{F}_{m{^{\prime}}}-F}{{F}_{m{^{\prime}}}}$$

After measurement of Y(II), far red light is illuminated to open the reaction centers, which are closed for measuring F_m′_. In a typical induction curve, the Y(II) measurements are cycled 12 times to determine the average value. This mean value obtained in this light adapted stage gives the effective light absorption capacity or effective yield of PS-II reaction center.

## Results and discussion

### Soil water retention and stomatal conductance response to drought stress

**Figure 4:**
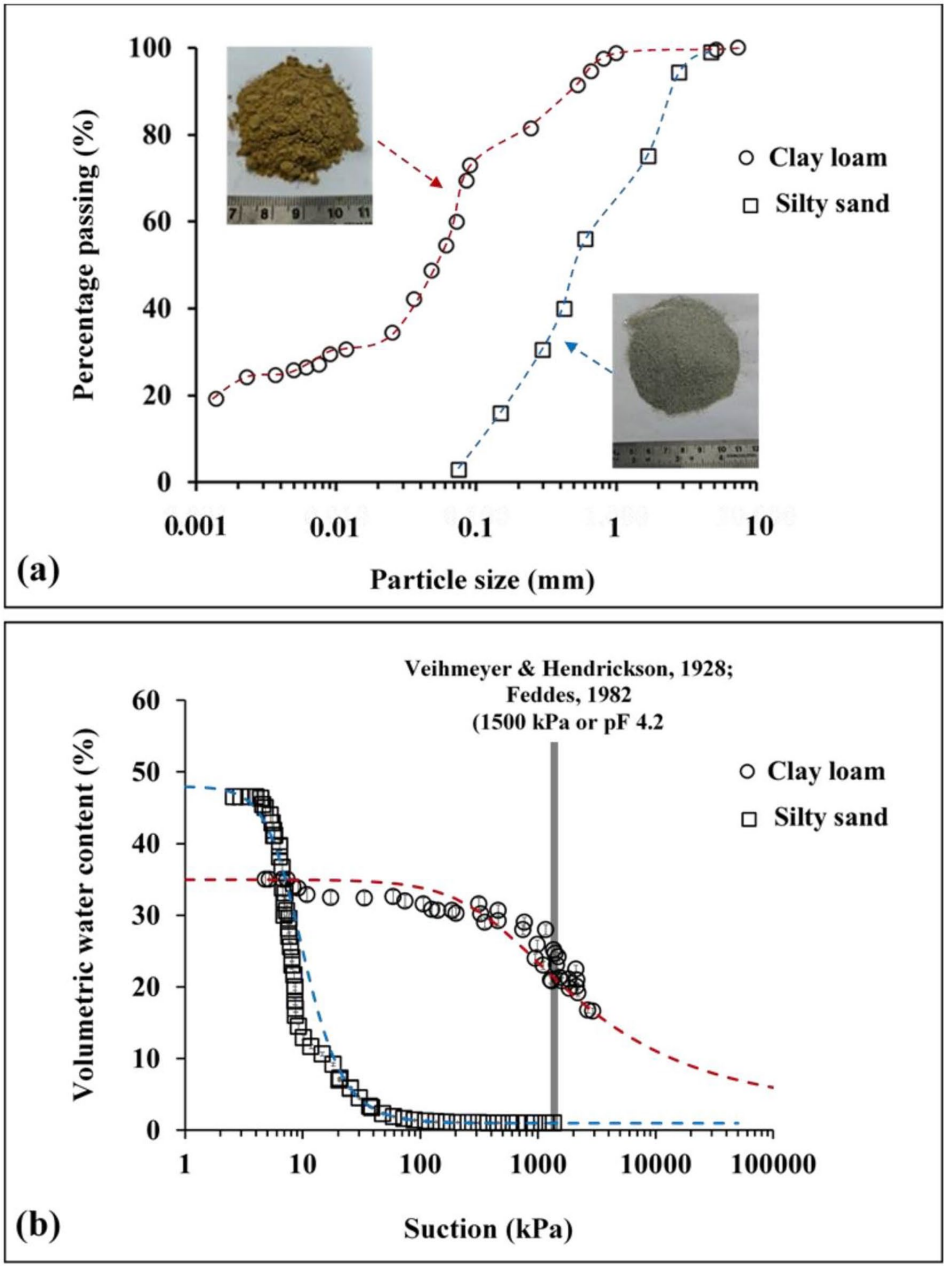
(**a**) Particle size gradation of the selected soils showing higher number of retained particles ranging between 0.001 and 0.01 mm for clay loam, and 0.1–1 mm for silty sand; (**b**) soil water characteristic curve of vegetated soil columns depicting higher retention at clay loam than silty sand. Also, the matric suction of 1500 kPa determined by Veihmeyer and Hendrickson (1928) and Feddes (1982) is marked to represent the traditionally considered permanent wilting point.

**Figure 5:**
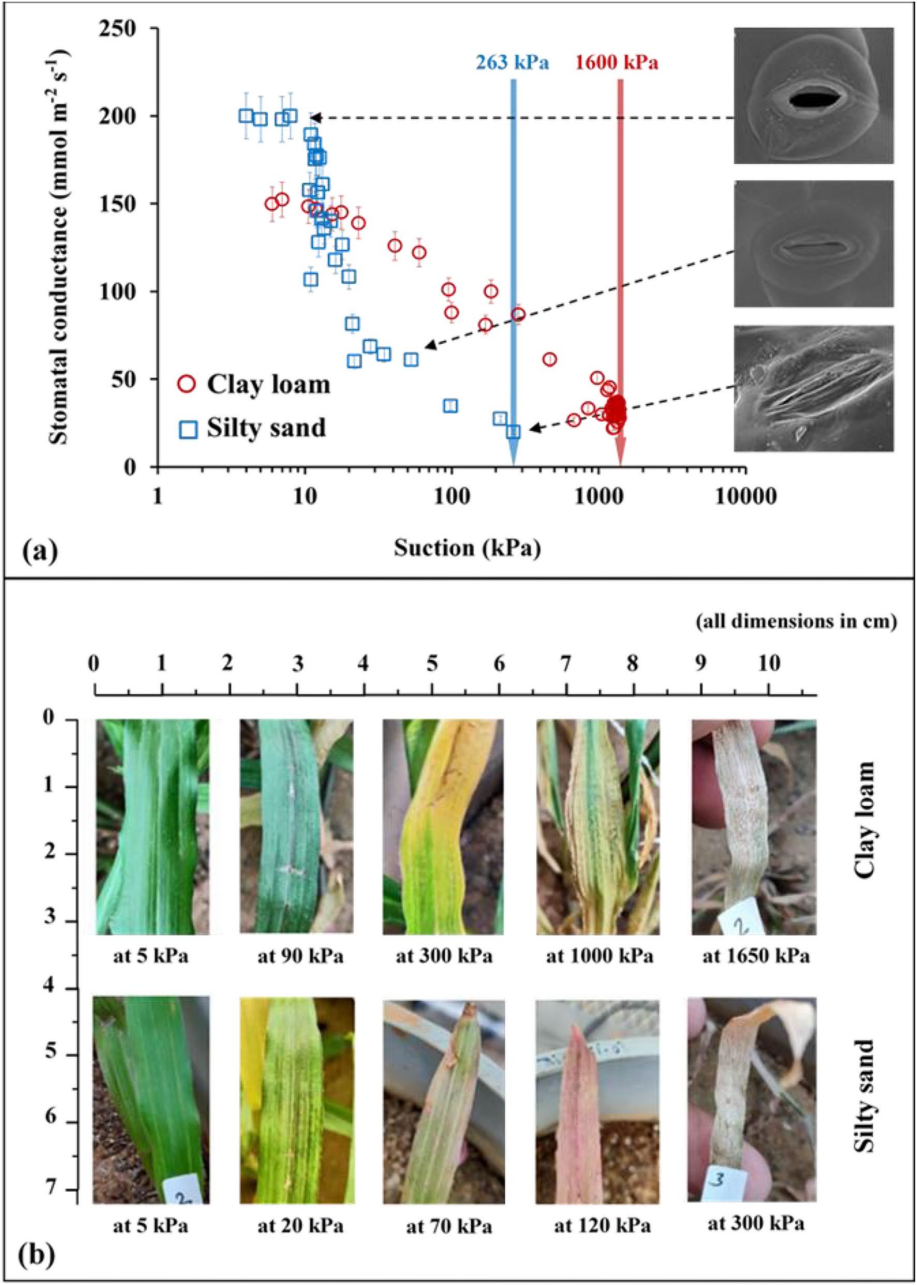
Drought response of vegetated soil columns presented by (**a**) stomatal conductance with the wilting point determined at both clay loam and clay loam (i.e. 1600 kPa and 263 kPa respectively). Also, the stomata images at different drought progressions are shown; (**b**) pictorial images of leaf under continued drought stage at different suction stresses.

**Figure 6:**
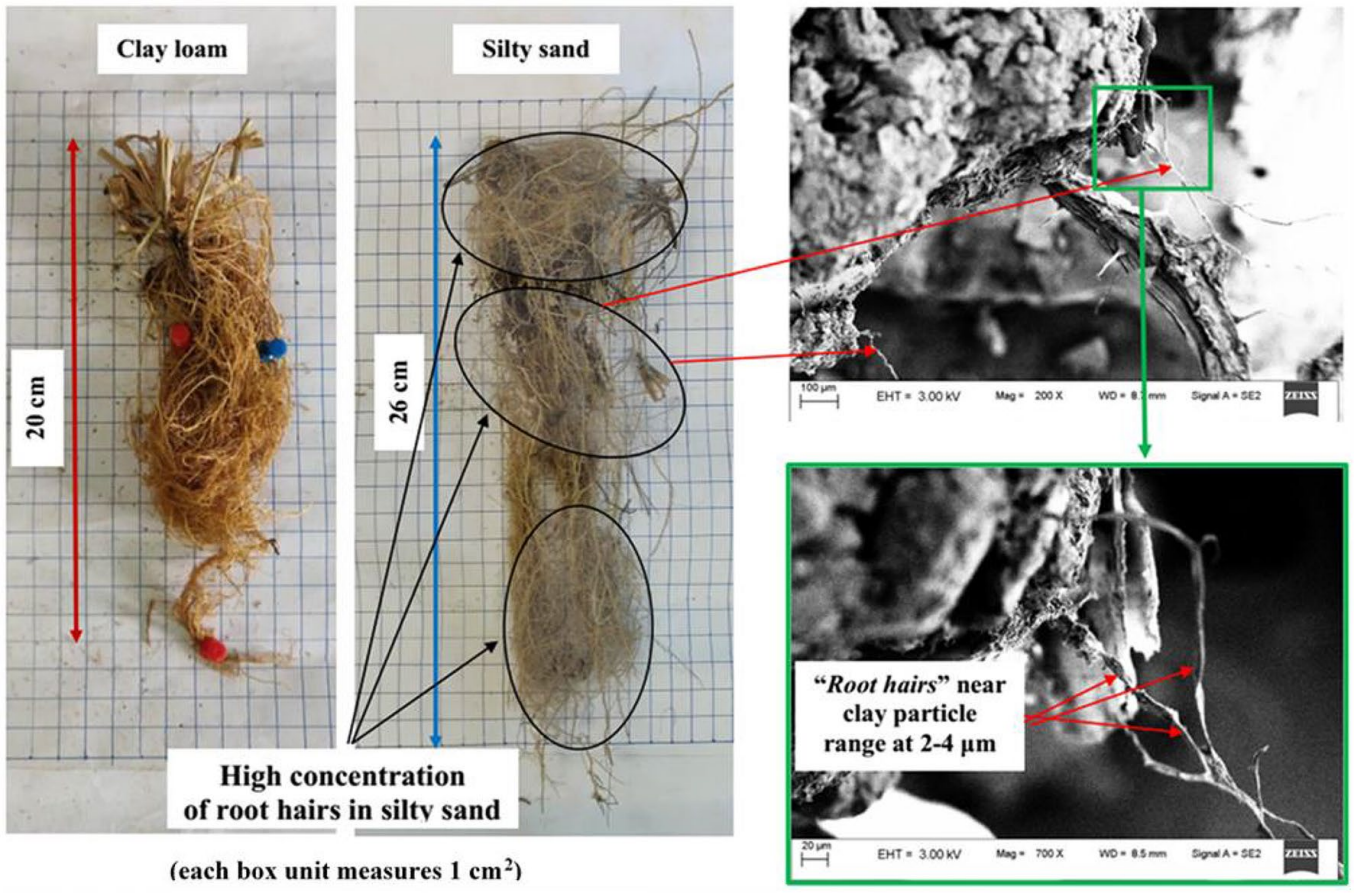
Pictorial description of excavated roots of the grass species depicting root length and fine “root hairs”.

Figure 4a,b presents the particle size distribution and SWRC of silty sand and clay loam from 4 kPa (pF 1.1) corresponding to saturated water content (${\theta }_{s}$) till 3000 kPa. In the present study, the sandy soil used consists of 98% fine sand (refer Table 1) and holds a hydraulic conductivity value of 10^–5^ m/s (calculated from falling head test). Based on the hydraulic conductivity values, it can be inferred that water will not drain quickly as expected for typical coarser sands^50^. It was observed that clay loam soil could retain more water than silty sand for the entire suction range. The ${\theta }_{s}$ for silty sand is initially higher at lower suction (1–7 kPa) as it is coarse grained in nature having higher porosity at the same soil compaction/density. Silty sand imposes less suction stress on roots (i.e., negative pore water pressure, which impede root water uptake for a particular decrease in moisture content) due to its coarse-grained nature. This is reflected by the SC response to incremental drought stress, as shown in Fig. 5a. The maximum SC at the start of the drought period was higher for vegetation grown in silty sand as compared to clay loam. In fact, silty sand having higher porosity will also have a higher concentration of dissolved O_2_ in comparison to clay loam. The maximum SC observed at the start of the drought period can be possibly explained based on root density and root depth. Figure 6 shows the root architecture of *Axonopus compressus* for both soil types. In the case of silty sand, roots are well distributed and proliferate throughout the soil depth having higher access to plant available water as compared to clay loam. In this case, root growth was concentrated and primarily at shallow depths. It is reported in literature^51^ that the water use efficiency (i.e., the transpiration rate) is low for concentrated roots. Since transpiration efficiency and SC are positively correlated^52^, it is safe to state that a uniform root system would lead to higher SC. Regardless, SC decreases with an increase in matric suction for both soil types, indicating gradual closure of leaf stomata and gradual cavitation in the xylem tissues leading to vegetation mortality^19^. SC reaches a limiting value (near 10 mmol m^−2^ s^−1^) when the plant reaches PWP marked by full closure of stomata (Fig. 5a). Upon drought stress, stomata gradually respond to chemical signals such as abscisic acid produced by dehydrating roots and close the stoma area, while the leaf water status is maintained^22^. The unique PWP for clay loam and silty sand was observed at near 1600 kPa and 300 kPa, respectively, as marked by the residual SC values (around 25 mmol m^−2^ s^−1^) and leaf color (yellowish brown). As transpiration ceases due to the gradual stoppage of root water uptake, the chlorophyll pigment (indicated by green color) diminishes (Fig. 5b). A similar decrease in chlorophyll concentration was measured for oak species (*Quercus ilex* and *Quercus suber)* with lower water content and increased leaf water potential due to continued drought^53^.

### Leaf photosynthesis response to drought stress

**Figure 7:**
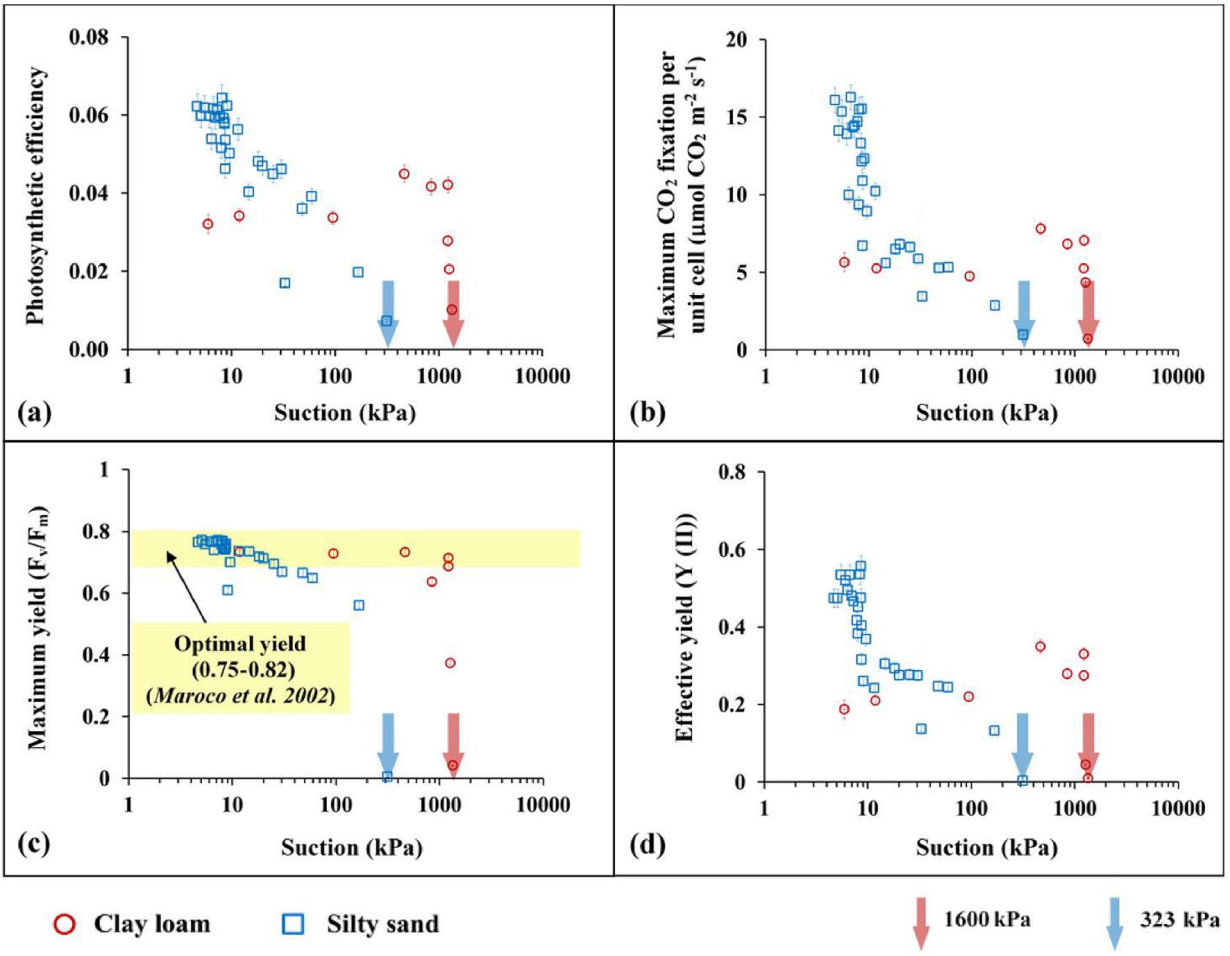
Photosynthetic parameters (**a**) photosynthetic efficiency; (**b**) maximum CO2 fixation rate per unit cell; (**c**) maximum yield; (**d**) effective yield vs suction plots under drought progression. The permanent wilting points determined at all the parameters are observed as 323 kPa and 1600 kPa for silty sand and clay loam, respectively.

Figure 7 presents the variation of photosynthetic parameters (photosynthetic efficiency, maximum CO_2_ fixation rate, maximum yield, and effective yield) with increased drought stress (suction). In both soils, the photosynthesis parameters cease to zero at distinct PWP (300 kPa for silty sand and 1600 kPa for clay loam), as seen in Fig. 7. However, the plant photosynthesis response to drought is different for both soil types due to the influence of water uptake in photosystem-II mechanism. Quantitatively, photosynthetic efficiency and CO_2_ fixation rates of vegetation were found to be higher for silty sand as compared to clay loam. This is specifically evident at lower suction values (≤ 20 kPa), wherein the moisture status of silty sand is much higher than loamy soil (refer SWRC, Fig. 4b). For silty sand, there is a gradual decrease in both these parameters, indicating that photosynthesis gradually decreases in the considered suction range. In contrast, for clay loam, the lower light use efficiency for photosynthesis can be attributed to the down regulation of C-metabolism, understood to be an adjustment of the photosystem (PS)-II machinery^54^.

The down regulation of C-metabolism is pronounced in clayey loam due to its relative low CO_2_ fixation rate. The lower photosynthetic efficiency and effective yield upon drought are particularly true for clay loam as the held water (due to charged clay minerals) within the soil matrix. This held water cannot be easily taken by relatively coarser root morphology developed in the soil (Fig. 6), thereby reducing the photosystem-II mechanism in the absorption of light. On the other hand, fine clusters of root hair formation in silty sand facilitate easy withdrawal of free water even at extremely low soil water content (2–4%; refer Fig. 4b). As soil undergoes gradual desaturation with available soil O_2_, the photosynthetic parameters (efficiency, CO_2_ fixation, and effective yield) of the plant gradually decrease with suction until it drastically drops at near PWP (≈ 1600 kPa). Silty sand, even though personifies low water retention, facilitates easy growth of root hairs, can adsorb dissolved O_2_ and water even at low suctions. Thus, plants in silty sand showed higher photosynthesis (higher high absorption in photosystem-II) (Fig. 7). Before the plant wilts (around 300 kPa), the maximum yield is at the optimum stage^55^.

### Root architecture influence on plant water availability and survival time during drought

**Figure 8:**
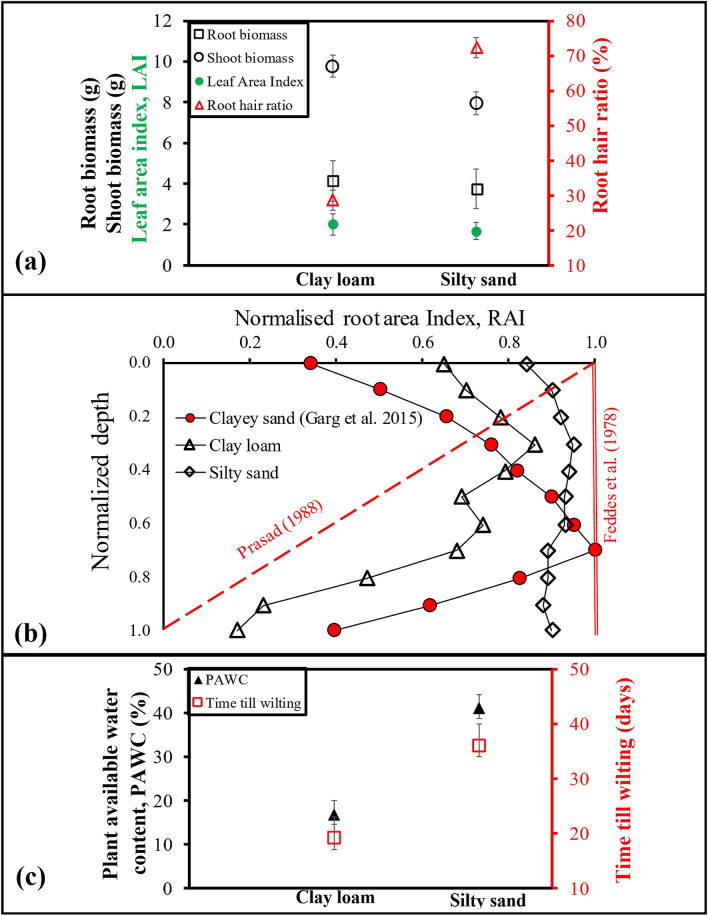
(**a**) Dry root and shoot characteristics of the vegetation in the tested soils. (**b**) Root architecture of the vegetation presented in the form of normalized root area index (i.e. Column area/Root area). (**c**) Plant available water content and time till wilting for A. compressus.

Figure 8a presents the above ground (shoot biomass and leaf area index (LAI)) and below ground (root biomass and root hair to total root biomass ratio) vegetation parameters after the end of the plant wilting. Shoot biomass was higher by 25% for clay loam in comparison to silty sand, while, for root biomass, there was almost equal growth in both soils. The LAI was also comparatively the same for both soils. However, the ratio of root hair (diameter less than 20 μm) mass to total root mass for silty sand was 2.6 times higher than clay loam. This facilitates easy capillary root water uptake from the inter-particle void in the case of silty sand^56^. The root distribution profile in silty sand is uniform, as indicated by the normalized root area index (RAI), whereas in clay loam, it tapers down with normalized depth (Fig. 8b). The uniform distribution of root hairs throughout the soil matrix helps withdraw water when the plant is water-stressed and contributes to the survival of the plant. The root distribution patterns assumed in crop models such as Prasad^﻿57﻿^ and Feddes et al.^58^ do not accurately capture the effect of root distribution while predicting the plant available water content (PAWC). Time till wilting after drought application was evidently higher for silty sand at an average of 38 days as compared to clay loam at 20 days. Comparing the soil water content corresponding to the wilting point obtained from the measured SC values and photosynthetic parameters in the current study, the PAWC was presented in Fig. 8c. Even though retention is lower for silty sand, the PAWC was three times higher than clay loam.

## Summary and conclusions

This study re-looks into the concept and definition of permanent wilting point (PWP) of a non-crop grass species subjected to drought, in two contrasting soils. This was done by integrating the measurements of soil water retention and plant leaf physiology (leaf stomatal conductance (SC) and photosynthesis parameters). Drought stress was quantified by measuring soil matric suction from field capacity to PWP and was related to the SC and photosynthetic parameters (photosynthetic efficiency, maximum CO_2_ fixation rate, maximum photosynthetic yield, and effective photosynthetic yield). Vegetation grew for 60 days on six instrumented columns (three for each soil type) for root and shoot establishment in an atmospheric controlled greenhouse. Thereafter, by keeping the shoot biomass constant, drought was applied by not irrigating all six columns until all vegetation wilted. The study presents a novel framework for obtaining the actual PWP by relating the photosynthesis parameters with soil matric suction. The role of root architecture in resisting drought conditions for a species type was also demonstrated.

The study provides experimental evidence based on soil and plant characteristics for the fact that PWP cannot be determined from a unique reference suction of 1500 kPa. A coarse textured soil causes wilting at much lower suction than the fine textured soil (around 300 kPa for silty sand and 1600 kPa for clay loam). Plant response to drought is dependent on the relative porosity and mineralogy of the soil, which govern the relative ease at which roots can assimilate O_2_ and held water, respectively. The PWP investigated from the variation of photosynthetic parameters with matric suction revealed that vegetation wilts at around 300 kPa and 1600 kPa for silty sand and clay loam, respectively. At low suction range for silty sand, photosynthetic efficiency and CO_2_ fixation rates decrease rapidly in magnitude. Clay loam shows lower photosynthetic efficiency and CO_2_ fixation rate (in magnitude with respect to silty sand) attributed to the down regulation of C-metabolism, understood to be an adjustment of the photosystem (PS)-II machinery. In the aspects of root water uptake, for clay loam, the held water (due to charged clay minerals) within the soil matrix, do not facilitate easy uptake of water by relatively coarser root morphology. Contrastingly, fine (2–17 μm in diameter) root hairs formation in silty sand aided higher plant water uptake and plant survival time (twice that of clay loam). The variable nature of the PWP and contrasting root architecture for the two soils indicate that crop models should incorporate the heterogeneity associated with soil type for developing irrigation schemes.

## Future scope

The current study only incorporates two types of soil to investigate the heterogeneity of the wilting point. Future studies need to be conducted on a range of soils to explore the range of PWP that might be expected in the field. The current study uses only one non-crop species to demonstrate PWP variability. Future studies incorporating crop species (especially sensitive to drought) with data relating changes of photosynthetic efficiency and C-metabolism pathways need to be conducted to understand the drought and PWP responses.
